# What is prepared in temporal preparation? A review and a historical appreciation

**DOI:** 10.3389/fpsyg.2026.1768431

**Published:** 2026-06-04

**Authors:** Karin M. Bausenhart, Rolf Ulrich

**Affiliations:** Department of Psychology, University of Tübingen, Tübingen, Germany

**Keywords:** foreperiod effect, locus, review, temporal attention, temporal orienting, temporal preparation

## Abstract

Temporal preparation refers to systematic changes in performance that arise when the timing of an event becomes predictable. From very early on in experimental psychology, research has shown that temporal predictability robustly shortens response times and modulates attentional states across a wide range of tasks. This review provides a comprehensive historical and contemporary synthesis of theoretical and empirical contributions to temporal preparation research. First, we present a brief overview of basic concepts, paradigms, and findings in temporal preparation research, and review mechanisms that have been suggested to address the question of how preparation emerges and how it may impact human cognitive processing. Second, in the main part of this article, we review findings from behavioral and electrophysiological studies that shed light on which cognitive processes are affected by temporal preparation in humans, aiming to take stock of the most robust empirical phenomena. Here, the main focus will be on the rich tradition of foreperiod effects, complemented by relevant results from temporal cueing and related paradigms. We show that temporal preparation has widespread consequences for cognitive processing, from increasing sensory gain to changes in response dynamics and various motor-related measures. These findings challenge traditional accounts that locate temporal preparation in specific processing stages (e.g., historically, the motor system). Instead, contemporary models must take into account a multi-component view of temporal preparation, in which preparatory processes impact various processes.

*By no means are we capable of tensing our attention evenly at all times*

(Wundt, 1862, p. 264, author's translation).

Already noted by Wilhelm Wundt at the very beginning of experimental psychological research, attentional fluctuations across time have since then been shown to affect various aspects of human information processing. Woodrow (1914) was the first to demonstrate that a warning signal (WS), temporally preceding the occurrence of an imperative stimulus, can greatly alter our state of attentiveness at the moment of stimulus presentation. In fact, it is now well established that reaction times are markedly accelerated when an imperative stimulus is made more temporally predictable through uninformative WSs, explicit temporal cues, or rhythmic entrainment. Numerous publications have addressed the mechanisms underlying and the consequences of this so-called *temporal preparation*.^1^ This paper provides a comprehensive review of this phenomenon, with an especially strong focus on the rich empirical tradition and classic literature regarding foreperiod effects, yet complemented by contemporary evidence from related paradigms and approaches.

In the first part, we introduce relevant terminology and common experimental paradigms used to investigate temporal preparation, including variants of the historically employed foreperiod paradigm and more novel approaches employing temporal cues, instructions, and rhythms. The roles of (implicit or explicit) temporal expectations and temporal uncertainty are illustrated, and it will be described how a variety of specific factors modulate temporal preparation. For example, even in the basic foreperiod paradigm, temporal preparation depends on a variety of characteristics of the WS and the imperative signal, as well as on their temporal relation.

Such characteristics may implicitly or explicitly affect the expectancy of when the target stimulus occurs: the less predictable the timing of the upcoming event, the less temporal preparation, and hence, the longer the response time (RT). Hence, the most prominent theoretical accounts of temporal preparation are outlined, including expectation-based and dual-process accounts (e.g., Vallesi and Shallice, 2007) as well as trace conditioning and related accounts (Los and Van Den Heuvel, 2001; Los et al., 2001, 2014; Salet et al., 2022), which predominantly deal with how preparatory states build up in time, and, finally, the early onset model (Rolke and Hofmann, 2007) and the motor readiness model (Mattes and Ulrich, 1997; Näätänen, 1971), which rather emphasize the locus of temporal preparation effects.

In the second part, the focus is on various behavioral and electrophysiological measures commonly used to assess temporal preparation. For several decades following Woodrow's pioneering work, the bulk of this empirical evidence documented preparation-induced changes in the motor system. For example, studies measuring reaction times (e.g., Sanders, 1980; Spijkers, 1990), response force (e.g., Mattes and Ulrich, 1997; Van der Lubbe et al., 2004), reflex amplitudes (Brunia et al., 1982; Requin et al., 1977; Zeigler et al., 2001), and corticospinal excitability (e.g., Davranche et al., 2007; Hasbroucq et al., 1997) led to the widespread belief that temporal preparation facilitates processing of imperative signals predominantly within motor processing stages. Other measures investigating perceptual or decisional stages of processing have traditionally received much less attention—notable early exceptions are, for example, Klein and Kerr (1974), Lowe (1967), and (Treisman and Howarth 1959). More recently, however, this gap has been addressed extensively through chronometric, psychophysical, and electrophysiological approaches, and a number of studies clearly point to pre-motor effects of temporal preparation across different modalities and several paradigms (for overviews, see also Bausenhart, 2009; Rolke and Ulrich, 2010). For example, temporal preparation has been shown to speed up processing in pre-motor processing stages (e.g., Bausenhart et al., 2006; Müller-Gethmann et al., 2003; Seifried et al., 2010) and to enhance perceptual analysis of visual and auditory stimuli (e.g., Bausenhart et al., 2007, 2008; Correa et al., 2005; Lange et al., 2003; Rolke and Hofmann, 2007; Rolke, 2008). In sum, temporal preparation may exert its effects at multiple stages of processing, particularly when demands within a given stage are high (cf. Correa et al., 2006a). This perspective may help reconcile the widespread and seemingly inconsistent effects of temporal preparation on cognitive performance.

## Basic concepts and experimental paradigms

Anticipating upcoming events helps us navigate a constantly changing environment and respond quickly and flexibly. Prior knowledge, experience, reasoning, and learning about how events follow one another enable expectations about upcoming situations. These expectations can shift our motivation, thoughts, and emotions, thereby influencing how we perceive things, set goals, choose among options, and learn (e.g., Sanders, 1966). As Requin et al. (1991) put it, every action grows out of a series of “antedating processes” (p. 358). If we have sufficiently precise expectations about an upcoming event, these mental steps may even occur before the event that requires a specific action. But expectations are rarely perfectly valid: we usually face a certain degree of uncertainty about what will happen (*event uncertainty*) and when it will happen (*temporal uncertainty*). For example, waiting at a traffic light mostly involves temporal uncertainty (when will it turn green?), whereas driving around an obscured curve entails event uncertainty (will the road be clear, or will the lane perhaps be blocked by another vehicle or something else?). Stronger, more valid expectations reduce these uncertainties and, in turn, allow preparatory processes, leading to more efficient actions.

For example, when event uncertainty is low, people can begin to preselect or preprogram an appropriate action. Because preparation involves “performing in advance what can be performed in advance of a response” (Näätänen and Merisalo, 1977, p. 133), providing more information about the upcoming event enables more preprocessing, and thus shortens RT (Requin et al., 1991). To examine how much preparation occurs before stimulus onset, researchers may vary a precue's informational value. Reducing event uncertainty—for example, by limiting the number of possible response alternatives—allows partial preparation of the appropriate response. In a classic study, Rosenbaum (1980) asked participants to move their hand toward a goal defined along three dimensions (hand, movement direction, movement distance). Cues revealed none, one, or several of these dimensions; the more information the cue provided, the more aspects of the target movement could be prepared in advance, and the shorter the RTs. Similar effects have been observed in many other studies (Goodman and Kelso, 1980; Hasbroucq et al., 1999b; Miller, 1982a; Osman et al., 2003; Rosenbaum, 1983).

But what can be prepared if we can only cast reliable predictions about the timing of an (otherwise unknown) upcoming event? Typical studies investigating this so-called “temporal preparation” use WSs or cues but don't inform participants what the upcoming target will be—they only provide information regarding the time of its occurrence.^2^ Even if the target requires a forced-choice response (i.e., event uncertainty is high), temporal uncertainty drops and temporal preparation effects emerge, typically facilitating performance (e.g., RT is shortened).

How can WSs that contain no explicit timing information nevertheless induce temporal preparation? Presumably, people acquire and make use of known relationships between WS and target—for example, an orange traffic light that usually turns green after a specific interval, or the rhythmic “ready, steady” before a starter's “go.” Once such a WS appears, we automatically begin preparing to respond quickly. Since Woodrow (1914), many studies have examined how these implicit WSs affect RT. Typically, such signals provide temporal information only through their *foreperiod* (FP), the temporal delay until the target appears. Manipulations of this delay allow researchers to study the build-up of temporal expectancies. Classic work has shown that FP characteristics such as duration and variability strongly shape temporal preparation (Bevan et al., 1965; Klemmer, 1956; Mattes and Ulrich, 1997; Müller-Gethmann et al., 2003). In general, less uncertainty about when the target will appear allows for more preparation and thus shortens RT.

### Foreperiod paradigms

The basic FP paradigm presents a WS, then after the FP, the target appears and participants respond. In simple-RT tasks, where the required response is always the same, people can fully prepare the response in advance (for an overview, Niemi and Näätänen, 1981). However, even when the WS is uninformative about the upcoming stimulus (and consequently, the required response), it shortens RT compared to trials without a WS (Bertelson, 1967; Bertelson and Tisseyre, 1969; Broadbent and Gregory, 1965).

This paradigm has been used in many variations, using different WSs (e.g., Bertelson and Tisseyre, 1969; Davis and Green, 1969; Rodway, 2005), different durations or ranges of FPs (e.g., Bertelson and Tisseyre, 1969; Drazin, 1961; Elliot, 1973; Karlin, 1959; Klemmer, 1956; Müller-Gethmann et al., 2003; Woodrow, 1914), different distributions of FPs (e.g., Bevan et al., 1965; Klemmer, 1956; Mattes and Ulrich, 1997), and different target probabilities (e.g., Buckolz and Rodgers, 1980; Drazin, 1961; Näätänen, 1972; Näätänen and Merisalo, 1977). All these factors have been shown to affect temporal preparation and, in turn, RT.

**Figure 1:**
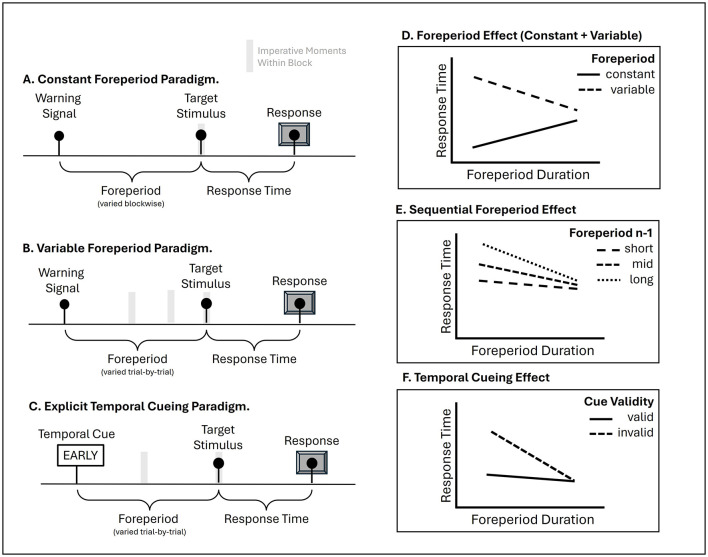
Common paradigms and typical result patterns in the study of temporal preparation. **(A)** Constant foreperiod (FP) paradigm: FP duration is constant within a block of trials and only varies between blocks. **(B)** Variable FP paradigm: FP duration varies from trial to trial, possible imperative moments are illustrated with gray vertical bars. **(C)** Explicit temporal cueing paradigm: Temporal cues indicate the duration of the upcoming FP either validly or invalidly. Here, an invalid temporal cue is illustrated. **(D–F)** Typical reaction time (RT) patterns in the depicted paradigms. For illustrative purposes, simple linear FP-RT relationships are depicted, even though non-linear and even non-monotonic trends have been observed under specific conditions (see main text). **(D)** Typical RT pattern in the constant and the variable FP paradigm: RT typically increases with FP for constant FPs and decreases with FP for variable FPs. **(E)** Sequential FP effects in the variable FP paradigm—especially at short FPs in the current trial *n*, the FP of trial *n* − 1 affects RT. **(F)** Typical RT pattern in temporal cueing studies: especially at shorter FPs, valid temporal cues produce shorter RT than invalid ones.

For example, Klemmer (1956) varied the range of FPs between blocks of trials but kept the mean FP constant across blocks. Even when only comparing trials with identical FP duration (i.e., those trials with exactly the mean FP duration), RT in those trials increased with increasing within-block variability of the FPs. Presumably, wider ranges of FP duration create weaker temporal expectations, thus reducing preparation and slowing responses. Absolute FP length also matters (e.g., Niemi and Näätänen, 1981). In the *constant FP paradigm* (cf. Figure 1A), where the FP duration is fixed within a block of trials, RT typically increases as the FP becomes longer (e.g., Karlin, 1959; Niemi and Näätänen, 1981; Woodrow, 1914, see Figure 1D). Longer delays induce greater temporal uncertainty: the longer one has to wait for the target, the less precisely one can predict its exact moment of occurrence. Very short FPs (up to roughly 200–400 ms), however, often show the opposite pattern: RT sharply decreases as FP increases up to this point, then follows the classic increase. This produces a U-shaped function (e.g., Bertelson and Tisseyre, 1969; Posner and Boies, 1971). Thus, very short FPs seem too brief for robust preparation, and benefits of such brief FPs may reflect arousal or intersensory facilitation effects rather than genuine preparatory processing (e.g., Hackley and Valle-Inclán, 2003; Semjen et al., 1973; Ulrich and Mattes, 1996, see also Box 1). Overall, no single optimal FP exists because the best timing depends on various task characteristics—for example, on properties of WS and imperative signal, as well as task difficulty (e.g., Niemi and Näätänen, 1981; Teichner, 1954).

Box 1 **Accessory stimulation and brief FPs.**

When FPs are short, the immediate enhancing effect of a WS on RT may be attributed to *intersensory facilitation* (Nickerson, 1973; Hershenson, 1962) rather than to genuine temporal preparation. This enhancing effect is typically observed when an accessory stimulus is presented temporally close to an imperative response signal. For example, in a focused-attention task, Giray and Ulrich (1993) instructed participants to make a speeded response as soon as they detected a visual imperative signal and to withhold their response if an auditory stimulus occurred alone. In some trials, however, the auditory stimulus was presented together with the visual stimulus. Although participants did not need to pay attention to the accessory auditory stimulus, RTs under bimodal stimulation were shorter than if the visual imperative stimulus was presented alone (275 vs. 314 ms), replicating earlier results (e.g., Bernstein et al., 1969; Morell, 1968; Posner et al., 1976).

This finding is also consistent with the *energy summation theory* of Bernstein (1970) that presumably was supposed to act at later processing stages than intersensory facilitation (see Giray and Ulrich, 1993). According to this theory, the activation elicited by the accessory and imperative stimulus is pooled in bimodal trials, producing greater coactivation than under unimodal stimulation. Yet, when the accessory stimulus is presented alone, the activation is insufficient to elicit a response. The notion that activation can be combined across response modalities has received substantial support from studies on the redundant-signal effect (Miller, 1982b; Schwarz, 1989).

This coactivation account may also explain why WSs that even briefly follow an imperative signal may nonetheless shorten RT (e.g., Leonhard et al., 2012; Miller, 1986). Some authors have used the terms “immediate arousal” (Sanders, 1999; Hackley and Valle-Inclán, 1999; Dufft and Ulrich, 1999) or “automatic alerting” (Hackley and Valle-Inclán, 1998) to describe shorter RTs for accompanying stimuli. This view is consistent with neuroanatomical results (for a review see Hackley, 2009). Accordingly, immediate arousal provides bottom-up activation that facilitates response elicitation processes.

In the *variable FP paradigm* (cf. Figure 1B), where FP duration varies from trial to trial, RTs are typically longer than in the constant version (e.g., Bevan et al., 1965; Klemmer, 1956; Mattes and Ulrich, 1997), with a reversed relationship regarding FP duration: RT decreases with increasing FP duration (e.g., Drazin, 1961; Hohle, 1965; Los et al., 2001, see Figure 1D). This pattern holds over a wide range of FP durations (e.g., Baumeister and Joubert, 1969; Elliot, 1973). Further refining this, it has recently been noted that the FP-RT function, when tested with sufficient FP range, may best be described as (non-linear) exponential decay function (i.e., with steeper RT decrease at shorter FPs than at longer ones, Houshmand Chatroudi et al., 2024).

Another robust finding in variable-FP tasks is that RT on trial *n* also depends on FP duration of the previous trial *n*−1 (e.g., Alegria, 1975a,b; Baumeister and Joubert, 1969; Drazin, 1961; Los et al., 2001, see Figure 1E). Fastest responses occur when a short FP is preceded by another short one, whereas a short FP preceded by a long one produces slower responses. These sequential FP effects are asymmetric, because they are strongest for the shortest FPs and vanish for the longest ones in a given block of trials (Drazin, 1961; Los and Van Den Heuvel, 2001; Vallesi et al., 2007; Woodrow, 1914, for a review, see Los, 2010). Interestingly, such sequential effects can be observed for both long (e.g., 1,200–3,600 ms) and short (e.g., 200–600 ms) FP sets (Steinborn et al., 2008), and even for higher-order sequences (i.e., going back more than one trial, Steinborn and Langner, 2012). Since averaging across these sequential patterns produces a pattern consistent with the typical variable-FP effect, it has sometimes been argued that trial-by-trial dependencies constitute a major source of the variable-FP effect. However, there is increasing evidence that other, longer-term components must also be involved (Capizzi et al., 2012; Los and Agter, 2005; Steinborn and Langner, 2011; Vallesi et al., 2013; Vallesi and Shallice, 2007, see also below).

For a more complete understanding of the sequential FP effect, note that a change in WS modality weakens it (Steinborn et al., 2009), and even subtle changes in the physical characteristics of an auditory WS can modulate this effect (Steinborn et al., 2010). Therefore, it appears that not only the duration of the preceding FP influences the temporal expectation of the imperative stimulus but also whether the current WS is consistent or inconsistent with the preceding WS. Although the sequential effect is weakened when the sensory modality or physical characteristic changes from the preceding to the current trial, it is not eliminated. This indicates the presence of modality-specific and amodal contributions to temporal preparation.

### Temporal orienting

Expectations about the timing of upcoming events can also be explicitly created using cues or instructions. Although the idea is old (McAdam et al., 1969), research interest in this type of preparation re-emerged in the last decades. In McAdam et al.'s classic study, participants predicted whether the upcoming FP would be short (1,200 ms) or long (2,400 ms). The experimenter then adjusted FPs to ensure that predictions were valid in 70% of trials (e.g., if the participant predicted the short FP, a short FP indeed followed with high probability). RTs clearly reflected these expectations: shortest for validly predicted short intervals, slightly longer for validly predicted long intervals, and longest for invalid predictions.

More recent temporal orienting studies typically investigate the effects of explicit expectations by using cues that directly signal the expected time of the target, such as the words “early” or “late,” or abstract symbols that are contingently paired with specific foreperiods and thereby gain predictive value (e.g., Correa et al., 2005, 2006b; Coull and Nobre, 1998; Miniussi et al., 1999, see Figure 1C). These cues can be valid or invalid, just as spatial cues in classic spatial orienting tasks (e.g., Henderson, 1996; Posner et al., 1980; Yeshurun and Carrasco, 1999), and participants may use them to temporally prepare. For example, Miniussi et al. (1999) observed faster responses when a short FP was validly cued (i.e., a “short” cue was followed by a short FP) than when it was invalidly cued (i.e., a “long” cue followed by a short FP); no such cueing effects were observed at the long FP (for an illustration, see Figure 1F). However, subsequent studies employing a higher proportion of catch trials (i.e., trials in which no target stimulus was presented) extended this effect to the long FPs in the respective range as well (Correa et al., 2004, 2006b). These results indicate that participants actively use temporal information provided by temporal cues to form temporal expectations and, in turn, adjust preparatory activity to the expected moment of target presentation. Interestingly, these effects are dissociable from variable-FP sequential effects (e.g., Capizzi et al., 2012, see also next section).

Instructions can also guide temporal expectations. Lange et al. (2003) used auditory stimuli separated by short (600 ms) or long (1,200 ms) intervals, varying randomly from trial to trial. Participants were instructed to respond only to louder, sparsely interspersed “deviant” tones when these were detected either after the short interval (in one block of trials) or the long interval (in another block of trials). This forced participants to attend specifically to the instructed moment. For both the rare deviant target tones as well as for the frequently presented standard tones (to which no overt response was required), various electrophysiological responses were enhanced when they were presented at the attended vs. the unattended moments, showing clearly that temporal attention can be successfully manipulated through instruction (see also Lange et al., 2006; Lange and Röder, 2006; Sanders and Astheimer, 2008).

Finally, presumably more implicit orientation of attention in time may also occur in the presence of rhythms. For example, target stimuli embedded in streams of regular (isochronous) or even within more complex temporal structure benefit compared to stimuli embedded within irregular or unlearned complex temporal structure (e.g., Cravo et al., 2013; de la Rosa et al., 2012; Heideman et al., 2016; Rohenkohl et al., 2012). Likewise, perceptual benefits arise for stimuli that are presented on- instead of off-beat within regular temporal sequences (e.g., Barnes and Jones, 2000; Jones et al., 2006; McAuley and Jones, 2003).

Especially the development of these latter paradigms has further established temporal preparation as a complex phenomenon with widespread effects throughout the cognitive system, and at the same time emphasized its close link to attention. Even though in the present article emphasis is put on the study of foreperiod effects, results pertaining to cue-, instruction- or rhythm-based temporal preparation will be included to complement and broaden the more classical field of foreperiod research. For excellent overviews on the conceptualization of temporal preparation as attention, and its relation and interplay with other attentional phenomena, such as, for example, spatial attention, see Capizzi et al. (2023), Nobre and van Ede (2023), and Seibold et al. (2023).

## What is prepared and when? Theoretical accounts

As outlined above, temporal preparation strongly affects performance. When people expect a stimulus at a particular moment, they respond more quickly. But reaching and maintaining an optimal preparatory state is effortful and cannot be sustained over long periods (e.g., Gottsdanker, 1975). This has motivated several accounts that try to explain when temporal preparation rises or falls. Below is an overview of the main theoretical ideas about when we prepare and what we prepare.

### Time uncertainty, hazard, and expectancy

FP effects are commonly explained in terms of *time uncertainty*, a view rooted in traditional models of time perception (e.g., Creelman, 1962; Klemmer, 1956; Treisman, 1964). For example, in constant FP designs, participants quickly learn the duration of the fixed interval and begin to prepare for the moment when it ends. But because time estimates are inherently imprecise, this preparation spreads over a “range of expected moments” (Treisman, 1964). In accordance with the *scalar property* of time, stating that the variability of time estimates increases proportionally with the duration being measured (thus implying a constant Weber fraction across durations), the “range” of expected moments increases approximately in proportion to the FP duration (Gibbon, 1977; Treisman, 1964). Longer intervals thus lead to wider ranges and greater uncertainty, which produces less precise preparation and longer RTs. Notably, when participants are instructed to synchronize their keypresses with the expected moment of target presentation, keypress distribution becomes increasingly variable as the FPs lengthen (Näätänen et al., 1974). Making the passage of time more traceable by providing countdown signals improves timing and thereby reduces RTs (Requin et al., 1991; Simon and Slaviero, 1975).

In variable FP designs, time uncertainty, of course, also increases with FP duration, yet an opposite pattern emerges in RT: RT decreases as the FP lengthens, indicating that time uncertainty alone cannot explain this result. Researchers have therefore suggested that *expectancy*, based on conditional probability and the concept of hazard rate (e.g., Elithorn and Lawrence, 1955; Luce, 1986; Näätänen and Merisalo, 1977; Niemi and Näätänen, 1981; Nobre et al., 2007; Trillenberg et al., 2000; Vallesi et al., 2013; Vangkilde et al., 2012) also modulates preparation in this case. Typically, FPs follow a rectangular distribution, meaning that each FP duration is equally likely at the start of a trial. However, as time within a given trial passes without a target onset, the conditional probability of target delivery increases. It has been suggested that participants use this pronounced increase in conditional probability (hazard) throughout the FP to build expectations and adjust their preparation, thereby overshadowing the increase of time uncertainty with increasing FP duration. Overall, this results in higher levels of preparation, and consequently shorter RT, at later time points in the trial (see Janssen and Shadlen, 2005, for a formal integration of time uncertainty, based on the Weber fraction, and build-up of anticipatory activity based on hazard rate). This explains why RTs are shortest at the longest FP, regardless of their absolute duration (Drazin, 1961), why manipulating the frequency of short vs. long FPs—using uneven or even non-aging FP distributions—can strongly reduce or even abolish the variable FP effect (Baumeister and Joubert, 1969; Frith and Done, 1986; Näätänen, 1970, 1971; Nickerson and Burnham, 1969; Trillenberg et al., 2000; Zahn and Rosenthal, 1966). Notably, the hazard-rate can also be tracked in electrophysiological measures, and located in the supplementary motor area (Herbst et al., 2018).

Even though the concept of hazard rate is commonly used to describe the buildup of temporal preparation, novel evidence suggests that the reciprocal event probability density function—mathematically simpler and more stable than hazard rate—may be better suited to model various RT patterns observed for different foreperiod distributions. Moreover, the effects of introducing catch trials may be captured through an independent component which can be formalized as an exponential function representing the influence of event uncertainty, which dynamically unfolds across foreperiod duration (cf. Grabenhorst et al., 2019, 2021). Remarkably, neural signatures of the the probability density function have recently been observed in parieto-temporal and sensorimotor cortical areas (Grabenhorst et al., 2025).

To explain sequential effects in the variable-FP design (see above), additional assumptions are required. That is, preparation is regulated not only by conditional probability for the specific FP in the current trial but also by expectations shaped by the FPs of preceding trials (Drazin, 1961; Karlin, 1959). Thus, a short–short FP sequence yields fast responses, whereas a long–short sequence slows performance. The asymmetry of sequential effects (little RT difference between short-long and long–long sequences) can then be explained by *temporal reorienting* (Alegria, 1975b; Alegria and Delhaye-Rembaux, 1975; Niemi and Näätänen, 1981): If an expected short FP ends without a target, participants can still shift preparation toward a later moment, thereby compensating for the lack of initial preparation at longer FPs. No such readjustment is possible when a target appears unexpectedly early.

Similar mechanisms may also underlie more recent temporal-orienting studies using explicit temporal cues. In analogy to spatial-attention studies (Posner et al., 1980; Henderson, 1996; Yeshurun and Carrasco, 1999), it is often assumed that valid cues direct attention to the expected moment of target onset, enhancing processing when expectation and reality align (see Seibold et al., 2023, for a recent overview focusing on the relation of temporal preparation and attention). The interplay of expectation and reorienting may also explain why temporal-cueing studies often fail to show cueing benefits at longer intervals—participants reorient toward the next imperative moment once the short interval has passed (e.g., Coull et al., 2000; Griffin et al., 2001; Los and Heslenfeld, 2005; Miniussi et al., 1999). Including a relatively high proportion of catch trials reduces the usefulness of reorienting as a strategy and thus restores cueing benefits at longer intervals (Correa et al., 2004, 2006b).

It should be noted that the sources and mechanisms underlying various temporal preparation effects may not be unitary. Even though sequential dependencies, temporal expectation (driven, for example, by time uncertainty and hazard rate) and temporal orienting or attention (driven by cues or instructions) all have been shown to improve performance, recent evidence suggests that these sources of preparation may be differentiated, and their interplay is complex. For example, Vallesi and Shallice (2007) proposed a *Dual-Process Model* that suggests different processes underlying the variable FP effect and the sequential FP effect. Specifically, the variable FP effect is assumed to reflect strategic monitoring of the hazard rate, while the sequential FP effect reflects a more automatic short-term component imposing trial-by-trial refractory costs. These costs reflect that preparatory resources may be depleted, especially after long FPs in the previous trial. This leads to a decreased state of arousal and thus to especially slow responses in the following trial when its FP is short (Vallesi et al., 2013, 2007). This dual-process view receives support from developmental (Vallesi and Shallice, 2007), anatomical (Triviño et al., 2010; Vallesi et al., 2007), and functional (e.g., Steinborn et al., 2010; Vallesi et al., 2014) dissociations of the variable-FP or temporal-orienting effects and the sequential-FP effect (note, however, that hazard-based explanations cannot readily account for long-term influences based on learning of foreperiod distributions (e.g., Los et al., 2017; Mattiesing et al., 2017, see also below).

Similarly, temporal orienting and hazard-based preparation may be dissociated: For example, Duyar et al. (2024) showed that validly cueing participants as to whether the first or second of two successively presented stimuli serves as target can increase perceptual performance and shorten RT, and this cueing benefit interacted with temporal uncertainty of the target timing (with largest benefits if target timing variability, and thus temporal uncertainty, was low). At the same time, the cueing benefit was reduced if the target stimulus was presented later than expected. That is, temporal attention, as directed by cues, was not re-directed quickly enough to track the increasing hazard-based target expectancy: attentional benefits declined even though hazard-based expectation increased (see also, e.g., Capizzi et al., 2012; Correa et al., 2014; Mento et al., 2015; Todorovic et al., 2015; Todorovic and Auksztulewicz, 2021, for related dissociations).

### Trace conditioning and multiple trace theory

While the suggestions outlined above encompass a multi-component view of temporal preparation, a competing explanation of FP effects suggests a single mechanism might underlie various preparation-related effects. This view proceeds from the notion of *trace conditioning* (Los and Van Den Heuvel, 2001; Los et al., 2001). Trace conditioning is a variant of classical conditioning in which a WS (CS) is followed—after a blank interval or “trace”—by a target (UCS) that elicits a response. With repeated pairings, the CS triggers a conditioned response (CR) that grows during the trace and typically peaks around the expected target time (Gallistel and Gibbon, 2000). In analogy to this, it can be assumed that the FP in typical FP paradigms serves as a trace interval, and the WS serves as a CS that automatically triggers a rise in response-related activation (Los and Van Den Heuvel, 2001; Los et al., 2001). In constant FP designs, learning stabilizes quickly and the activation peaks near target onset, with a higher asymptote of the peak at short and a lower asymptote after long traces, thus predicting slower RTs with longer FPs.

In variable FP designs, the model assumes learning at several “critical moments” (the possible target times). Moments reinforced by a target in trial n–1 gain activation on trial n, while bypassed moments undergo extinction. This produces the classic pattern: short RTs after repeated short FPs, slowing after long-short transitions, and little difference between short–long and long–long transitions. Indeed, the model reproduces both the variable FP effect and its sequential effects (Los, 2013; Los et al., 2001; Steinborn et al., 2008), and can also validly predict sequential FP effects even with high catch trial proportions (Capizzi et al., 2015, but cf. their discussion for an alternative suggestion in terms of repetition priming). However, more recent evidence shows that trace conditioning is not sufficient to account for the full richness of temporal preparation (e.g., longer-term preparatory adjustments).

Therefore, *Multiple Trace Theory of Temporal Preparation, MTP* (Los et al., 2014) extends the conditioning idea by proposing that each trial leaves behind a memory trace reflecting the accumulated strength of inhibition and activation at the critical moments, along with representations of the presented stimuli and responses. Whenever a new WS is presented, this acts as a retrieval cue for the previously established memory traces, which then contribute to the preparatory level in the current trial. As more recent traces are more activated, they contribute with greater weight to the preparatory profile than temporally more distant traces. This allows to account for a wide range of phenomena, including constant and variable FP effects, trial-by-trial and longer-range sequential effects (Steinborn and Langner, 2012), as well as “lingering” long-term influences of formerly experienced FP distributions (Crowe and Kent, 2019; Los et al., 2014, 2017; Mattiesing et al., 2017). MTP's recent successor model, the *Formalized Multiple Trace Theory of Temporal Preparation* (*fMTP*; Salet et al., 2022), provides a unified computational framework specifying how associative learning processes, driven by the interplay of temporal perception, motor activation and inhibition, and memory aspects, can account for a large range of empirical findings, notably without invoking explicit, controlled orienting processes based on expectancy and Hazard rate. Developmental, anatomical, and functional dissociations between sequential and variable-FP effects, as reported above, however, may be more difficult to account for in this unified framework.

### What is prepared? Early onset, motor readiness, and other

A central question in research on temporal preparation concerns in which stages of processing its effects arise. The *Motor Readiness Model* (Näätänen, 1971) proposes that excitatory and inhibitory commands fluctuate continuously in the motor system, and their difference defines motor readiness. When this readiness crosses a fixed motor action limit, a response is emitted. Importantly, motor readiness is actively regulated so that it approaches—but does not prematurely cross—the action limit. Increased temporal preparation reduces the distance between current readiness and the action limit, thereby enabling especially fast responses (Näätänen and Merisalo, 1977). Because this account locates temporal preparation effects at the motor level, it predicts changes in motor-related variables such as response force, which can be validated (Mattes and Ulrich, 1997). In addition, it can also account for premature responses if motor readiness is noisy and approaches the motor limit closely.

In contrast, the *Early Onset Model* (Rolke, 2008; Rolke and Hofmann, 2007) assumes that temporal preparation affects pre-motor processing. Based on criterion models of RT (Grice, 1968; Luce, 1986), it proposes that after stimulus onset, internal activation accumulates until a decision criterion is reached. Under good temporal preparation, this accumulation process can start earlier, so the criterion is reached sooner. When accumulation is interrupted—because responses must be very fast or stimulus information is cut off—higher temporal preparation allows more information to be gathered before interruption, leading to higher accuracy. Thus, this model predicts an early, perceptual locus of temporal preparation effects, contrasting with the motor locus assumed by the motor readiness model.

With regard to the expectancy- or conditioning-based accounts outlined above, it should be noted that these models emphasize the temporal dynamics of how expectancy profiles develop over the course of trials, and not necessarily tie temporal preparation effects to specific processing stages. For example, MTP and *f* MTP's computational framework (as outlined above) were originally devised to predict the interplay of motor activation and inhibition during the foreperiod, but in principle, they support the prediction of multi-stage effects. For example, the inhibition-activation units proposed by this model could also be applied to pre-motor, perceptual regions—thereby allowing for multiple loci of temporal preparation effects (Salet et al., 2022).

## Locus of temporal preparation effects: Evidence from various outcome measures

As outlined above, different accounts have been put forward to explain how and where, within the cognitive system, temporal preparation effects may emerge. Particular models are specific with regard to the question of *which* processes may be affected by temporal preparation (e.g., perceptual in case of the Early Onset Model, motoric in case of the Motor Readiness Model), however, these models are not necessarily mutually exclusive, and different mechanisms may in fact underlie preparation effects in different processing stages. Other models, such as fMTP, may explain more flexible preparation-related adjustments throughout the cognitive system within a single theoretical framework. In any case, it seems beneficial to systematically compile and analyze the potential effects of temporal preparation on various stages of human information processing. In the following, we will therefore review relevant empirical findings on the effects that temporal preparation exerts on various behavioral and electrophysiological measures, with respect to how they inform about potential loci within the processing system.^3^ It should be noted that this review does not aim to provide decisive evidence for or against one or the other theoretical account of temporal preparation (even though we will on occasion describe interpretation within a given theoretical framework where this may improve clarity). Rather, we intend to give a broad overview over the rich empirical literature pertinent to this question. Hopefully, this will lay the groundwork to foster further theoretical advances and/or refinements where they may seem in order. Specifically, we will review behavioral evidence coming from the study of RT, response force, and perceptual sensitivity, as well as electrophysiological evidence focusing on electromyographic activation and reflex amplitudes, eye movements, effects of transcranial magnetic stimulation, and EEG components, such as the Contingent Negative Variation, the Lateralized Readiness Potential, and other Event-Related Potentials.

### Response time

RT reflects the summed duration of multiple processing stages, so an RT effect alone cannot reveal where temporal preparation exerts its influence. However, various methods based on established RT models have been developed that may help identify such loci. For example, the *additive factors method* (Sternberg, 1969) assumes a sequence of independent processing stages: perceptual identification, central decision/response selection, and motor programming/execution. This model predicts that factors acting on different stages should combine additively, whereas factors producing interactions should operate on the same stage. Thus, when the locus of one factor is known, additivity or interaction with another factor can indicate whether they share a common locus.

Sanders (1980) applied this logic to FP effects by manipulating instructed muscle tension (tense vs. relaxed) and constant FP duration (1 vs. 10 s). RT and movement time were measured in a reaching task. He found that relaxing the muscles increased both RT and movement time, and that long FPs slowed RT relative to short FPs. Crucially, FP duration and muscle tension interacted. Specifically, the RT difference between tense and relaxed states was smaller for long than for short FPs. A second experiment replicated this pattern for frequent (55%) but not infrequent (15%) target stimuli, and a third experiment showed additive effects of muscle tension, stimulus–response compatibility, and signal degradation. From this pattern, Sanders (1980) proposed a multistage model, in which FP duration and muscle tension both act on the motor adjustment stage, linking an earlier motoric programming stage to the peripheral motor system. According to this model, earlier perceptual and response-selection stages are influenced by stimulus quality and stimulus–response compatibility, but not by the preparatory state, and stimulus–response frequency was assumed to influence several stages simultaneously.

However, later work questioned whether Sanders' tension effects truly reflected peripheral motor adjustment. Kimura et al. (2002) compared relaxed, self-selected tension (as in Sanders' study), and experimenter-imposed tension. Although muscle tension was equated between the two “tense” conditions, self-selected tension still produced the shortest RTs and pre-motor times, whereas motor time was unaffected. This suggests that the benefits attributed to “muscle tension” may stem from central attentional or preparatory processes rather than actual peripheral muscle state. Further studies nevertheless supported a motor locus. Spijkers and Walter (1985) and Spijkers (1990) found additive effects of FP duration and movement velocity (ascribed to motor programming), but interactions between FP and response-specific movement similarity (ascribed to the motor adjustment stage). Meulenbroek and Van Galen (1988), using a drawing task, localized FP effects to a movement-parametrization stage (one of three distinct motoric stages assumed by their model), based on interactions with movement length and additive effects with movement direction and number of elements.

Additional support for a motor locus comes from additivity between FP duration and variables known to affect perceptual or response-selection stages, such as stimulus quality (Frowein and Sanders, 1978), stimulus intensity (Bernstein et al., 1973; Niemi, 1979), WS intensity (Bernstein et al., 1973; Loveless and Sanford, 1975), number of response alternatives (Alegria and Bertelson, 1970), and stimulus-response compatibility (Frowein and Sanders, 1978; Posner et al., 1973; Sanders, 1977; Spijkers and Walter, 1985). Because these factors influence early or central stages, additivity suggests that the FP primarily influences later, motor stages. However, few studies provide exceptions. For example, Niemi and Lehtonen (1982) reported interactions between FP and visual or auditory intensity for variable (but not constant) FPs. Similar interactions with auditory (but not visual) target intensity (Niemi, 1979; Sanders, 1975), which are linked to early identification stages, could indicate perceptual involvement; however, intense auditory stimuli may also directly increase arousal (Bertelson and Tisseyre, 1969; Mattes and Ulrich, 1997; Sanders, 1977), complicating interpretation (see also Box 1). Lastly, Leonhard et al. (2012) examined the FP effects of auditory WSs that either preceded or followed a dim or bright visual response signal. Visual intensity modulated the FP effect, suggesting that at least some preparation operates at early visual stages.

A few studies even suggest a central locus. Simon and Slaviero (1975) found larger temporal preparation effects in a 2AFC task than in a simple RT task when FPs were filled with rhythmic time markers rather than empty, suggesting effects on stimulus identification or response selection. However, this relies on Donders' subtraction method (Donders, 1969), which may be criticized (Kuelpe, 1893; Luce, 1986). Broadbent and Gregory (1965) also reported an interaction between stimulus-response compatibility and temporal preparation in 2-choice responses, but this did not generalize to 4-choice responses and contradicts several other studies (Frowein and Sanders, 1978; Sanders, 1977; Spijkers and Walter, 1985).

Novel research re-investigated the role of temporal preparation in response selection. For example, a WS in the constant FP paradigm amplified the flanker effect at a 400-ms FP (Schneider, 2018a,b). With variable FPs, Weinbach and Henik (2013) observed a comparable increase at a 500-ms FP. Recently, Han and Proctor (2023) demonstrated that very short FPs (up to 200 ms) shorten RT, at the cost of increased error rates. This points to response criterion shift, where longer FPs may allow for improved information processing; additionally, at least without performance feedback, FP interacted with spatial S-R compatibility. Given the brevity of these FPs, however, these results are likely due to phasic alertness caused by the WS, and a more automatic, exogenous “mode of attention” rather than temporal predictability (Lawrence and Klein, 2013).

Overall, most additive-factor findings support a motor locus of temporal preparation, although some evidence points to perceptual or central contributions. The interpretation of these results is further constrained by criticisms of the additive factors method itself, particularly the assumption of strictly serial, independent processing stages (McClelland, 1979; Miller et al., 1995).

Subsequently, other chronometric methods have been employed to locate temporal preparation effects. For example, Bausenhart et al. (2006) employed the effect propagation property of the Psychological Refractory Period paradigm (e.g., Pashler, 1994), developed initially to account for performance deficits in dual-tasking, which allows dissociating effects on premotor vs. motor stages. They found constant-FP temporal preparation effects for a primary choice-RT task, and crucially, these propagated fully to a secondary task presented briefly after the first one (i.e., after a short stimulus onset asynchrony, SOA). No such carry-over was observed if the second task was presented after a longer SOA. This pattern corresponds exactly to what is predicted for the preparation effect emerging in the pre-motor stages of task 1 processing. Alternatively, with a motoric locus of the temporal preparation effect, the PRP model's cognitive architecture would have predicted no propagation of the preparation effect to task 2. In other words, these results indicate that temporal preparation influenced pre-motor processes exclusively.

Finally, some studies have also focused on preparation-related changes in response dynamics. For example, Bausenhart et al. (2010) combined a constant FP paradigm with a response signal method (Carrasco et al., 2006; Miller et al., 2008) to track the speed-accuracy trade-off function. Specifically, they presented a perceptually demanding visual target (a cross with one “leg” slightly longer or shorter than the other ones) after a short or long constant FP. Following the target, a response signal was presented at varying intervals from 50 to 2,000 ms, and participants were required to respond within a 300 ms time window after response signal onset. The resulting speed-accuracy trade-off function showed a typical pattern: especially fast reactions yielded discrimination performance at or close to chance level, followed by a sharp increase in accuracy with increasing response latency, which saturated at an asymptotic accuracy level for response latencies of about 800 ms or above. Importantly, only two of the three parameters that define this function were affected by the manipulation of FP: shorter FPs yielded an earlier intercept, indicating that performance departs earlier from chance level when participants can prepare better for target onset, and a higher asymptotic performance level, indicating that, given ample processing time, visual sensitivity reaches a higher level under conditions of good temporal preparation. The rate parameter, indicating the speed of information accumulation, was not affected by FP. These results are well in line with the predictions of the Early Onset Model, which assumes that temporal preparation leads to an earlier onset of information accumulation.

This finding was subsequently backed up by other studies: For example, Seibold et al. (2011) manipulated response criterion in a FP paradigm by introducing varying proportions of catch trials and observed that this manipulation produced additive effects with FP, which again suggests that temporal preparation affects the onset of information accumulation, but not the rate of this process. Consistently, Jepma et al. (2012) reported effects of constant FPs and temporal cues on the duration of non-decisional, but not on decision-related processes as threshold-setting and the rate of evidence accumulation (see also Devine et al., 2019; van den Brink et al., 2021, for converging neurophysiological evidence). In contrast, when using variable FPs and different hazard rates, Vangkilde et al. (2012) reported clear effects on the rate of information accumulation rather than on the onset of processing (see also Cravo et al., 2013; Rohenkohl et al., 2012, for similar results regarding expectations based on rhythmic entrainment, i.e., when targets were embedded within temporally regular or irregular streams of visual stimulation). While this suggests that the type of temporal information provided may differentially impact aspects of sensory processing, in general, these findings are well in line with an influence of preparatory processing at the perceptual level.

### Response force

Response force, as a motor-related measure, has repeatedly been shown to vary with temporal preparation (Giray, 1990; Jaśkowski and Verleger, 1993; Mattes and Ulrich, 1997). In a simple RT task, Mattes and Ulrich (1997) manipulated both FP duration (500, 1,750, 3,000 ms) and FP distribution (constant vs. variable). RTs showed the typical pattern: RT increased with constant-FP duration, and decreased with variable-FP duration. Mean RTs were also longer in the variable than the constant condition, reflecting reduced expectancy. Crucially, response force showed a similar pattern: responses were weaker when temporal preparation was high (short constant or long variable FPs) and stronger when preparation was low. Thus, temporal preparation appears to support not only fast but also energetically efficient responding.

A comparable dependency of force on FP was found by Van der Lubbe et al. (2004), although in their study force did not reflect the sequential modulations typically seen in RT. Consistently, Stahl and Rammsayer (2005) reported that the effects of an accessory stimulus on response force did not mirror its effects on RT, suggesting that speed and force may represent functionally independent components of sensorimotor processing. Generally, however, preparation-related effects on response force align well with the Motor Readiness Model (Mattes and Ulrich, 1997; Näätänen, 1971), stating that high preparation results in elevated baseline motor activation (motor readiness). Then, only small activation increments are needed to elicit a response, which will produce relatively soft and well-calibrated responses. Conversely, under low preparation, the system must generate a larger boost of activation to reach the motor action limit, which increases the likelihood of an overshoot and thus more forceful responses.

### Perceptual sensitivity

Compared with effects on RT measures or motor-related parameters, effects on perceptual sensitivity have been underappreciated in early work. In RT tasks, typically highly discriminable stimuli are used, and response speed is emphasized, so accuracy measured in these tasks often shows ceiling effects and inconsistent results. For example, some studies reported no effect of temporal preparation on accuracy (Alegria and Delhaye-Rembaux, 1975; Sanders, 1975), while others found improvements (Posner et al., 1973; Spijkers, 1990) or impairments (Bernstein et al., 1973; Bertelson, 1967; Bertelson and Tisseyre, 1969). Yet, improvements in accuracy in perceptually more demanding tasks may, in fact, indicate influences on perceptual processing.

Only few classical studies explicitly addressed this issue, mainly using detection tasks. Auditory detection studies demonstrated that FP and temporal expectancies modulate detection thresholds. Specifically, targets following shorter constant FPs or targets presented after more frequent FPs (hence at expected moments) were detected better (e.g., Howarth and Treisman, 1958, 1961; Loveless, 1975; Treisman, 1964; Treisman and Howarth, 1959; Wright and Fitzgerald, 2004); the pattern seems less clear, however, regarding variable FPs (Howarth and Treisman, 1958; Wright and Fitzgerald, 2004). Similarly, in the visual modality, preparation facilitates target detection. For example, weak targets, presented at random times during intervals of varying duration (i.e., different FP ranges), were detected best at intermediate intervals (Klein and Kerr, 1974; Lasley and Cohn, 1981; Lowe, 1967). Even though the exact pattern by which detectability depends on FP duration in these studies did not closely mirror the typical pattern observed in RT studies, these effects suggest that temporal expectancies may affect visual perceptual processing, possibly by improving information uptake or reducing internal noise (Macmillan and Creelman, 1991; Wickens, 2001). Alternatively, temporal preparation in detection tasks may also influence decision processes (Posner et al., 1973; Treisman, 1964). For instance, WSs could also facilitate “target present” decisions in perceptually ambiguous situations (e.g., Treisman and Howarth, 1959, observed reduced detection thresholds even for WSs presented after the target), and several studies did not report criterion measures that would allow to evaluate this claim. Consequently, the precise stage affected—perceptual or decisional—remains somewhat ambiguous in detection tasks.

More recent studies using discrimination tasks help clarify this issue. For example, valid temporal cues enhanced *d*′ for detection and discrimination of target letters presented within a stream of rapidly presented non-target letters, without affecting the decision criterion (Correa et al., 2005). Similarly, in backward-masked visual discrimination tasks, short constant FPs improved both RT and accuracy for the detection of rather simple (gap position in a Landolt square) and of more complex visual stimuli (letters), without changing the response criterion (Rolke, 2008; Rolke and Hofmann, 2007). Based on the claim that backwards masking selectively disrupts perceptual processing and visual memory traces (Kahneman, 1968; Sperling, 1963), these findings strongly support a perceptual locus of temporal preparation, particularly when perceptual demands are high. Interestingly, a recent study by van Ede et al. (2020) further supported this idea by comparing temporal cueing effects on RT and accuracy directly between a speeded response in a go/nogo task and a perceptually demanding task requiring orientation discrimination for masked Gabor patches. In line with the claim that the locus of preparation effects may flexibly depend on task demands, valid temporal expectations improved accuracy only in the perceptual task, whereas RT was shortened in both tasks. Finally, temporal expectation guided by rhythms improves visual processing, for example by enhancing contrast sensitivity (Cravo et al., 2013).

Beneficial effects of temporal preparation were also observed for auditory pitch discrimination, with shorter constant FPs leading to higher auditory sensitivity than longer constant ones (Bausenhart et al., 2007), constant compared to variable FPs (Herbst et al., 2022), and with implicit temporal cues (Herbst and Obleser, 2019). Interestingly, a study by Seifried et al. (2010) provided converging evidence for an influence on auditory information intake using the rotating spot method. Specifically, their participants watched a revolving clock hand, and reported its position when they detected an auditory signal. When temporal preparation, provided through constant foreperiods, was high, perceptual latency (as measured from the deviation of the perceived from the actual clock hand position at target onset) was reduced compared to conditions of lower temporal preparation, supporting the idea that temporal preparation diminishes the duration of perceptual processing also for auditory stimulation.

Temporal aspects of perceptual processing also seem to be affected by temporal preparation. For instance, explicit temporal cueing of the target appearance increases temporal resolution, as indicated by higher discrimination performance (i.e., smaller Just Noticeable Differences, JND) after valid compared to invalid temporal cues in a temporal-order judgment task (Correa et al., 2006c). This finding was later extended to the constant FP paradigm, where JNDs for temporal order judgment also varied with FP duration (Bausenhart et al., 2008). When measured across a large range of constant FPs, JNDs even followed the pattern typically observed for RT in constant FP designs—a steep initial decrease in JND (i.e., an increase in temporal resolution) at short FPs and a gradual increase in JND (i.e., decreasing sensitivity) with further lengthening of FP duration. Finally, at least within variable-FP contexts, perceived target duration is longer after long than short FPs, and sometimes more accurately reported (Bendixen et al., 2006; Grondin and Rammsayer, 2003; Mo and George, 1977). Interestingly, no such effects on duration could be observed when temporal preparation was induced with constant FPs (Grondin and Rammsayer, 2003; Mo and George, 1977). In sum, various preparation-related effects on perceptual sensitivity measures, as accuracy or sensory thresholds, suggest that perceptual processes can also directly benefit from temporal preparation.

### Electromyographic activation and reflex amplitudes

Electromyography (EMG) records muscle activation and allows RT to be partitioned into pre-motor time (stimulus–to-EMG onset) and motor time (EMG onset–to-response). Several studies used this decomposition to localize temporal preparation effects. Some studies found no effect of temporal preparation on motor time despite clear differences in RT (Botwinick and Thompson, 1966; Sanders, 1980), suggesting that preparation shortens pre-motor processes. Other studies, however, observed small but reliable reductions in motor time with improved preparation (Hasbroucq et al., 1995; Tandonnet et al., 2003). These changes were minimal relative to the overall RT effect. For example, Tandonnet et al. (2003) reported a 3-ms reduction in motor time vs. a 20-ms FP effect on RT, most of which occurred in pre-motor time. Thus, overall, EMG studies indicate that temporal preparation predominantly affects processes prior to peripheral muscular activation, but may also slightly influence motor execution. However, pre-motor time as indicated by EMG onset is not completely free of motor-processing components, as it includes perceptual, decisional, and centrally located motor programming stages. Therefore, EMG cannot unambiguously distinguish between pre-motor and motor loci of preparation.

Reflex studies provide another window into motor-level preparation by examining changes in spinal excitability. Brunia et al. (1982) elicited Achilles tendon reflexes in both legs during a fixed 4-s FP while participants prepared left and right foot or hand responses. Reflex amplitudes showed an early, nonspecific increase shortly after the WS—an arousal effect seen in both legs and even when no response was required (Scheirs and Brunia, 1985; Semjen et al., 1973). Throughout the FP, however, reflex amplitudes were lower in the response-involved leg than in the non-involved leg, consistent with motor-specific preparatory inhibition that prevents premature responding. This pattern was reversed after target onset, reflecting selective activation for response execution. Comparable modulations have been observed using tendon reflexes (Brunia, 1983; Brunia et al., 1982; Scheirs and Brunia, 1982), Hoffmann reflexes (Manning and Hammond, 1990; Requin et al., 1977), and eye-blink reflexes (Boelhouwer et al., 1991; Low et al., 1996; Sollers and Hackley, 1997; Zeigler et al., 2001).

Most of these studies, however, used a single fixed FP, limiting conclusions about how changes in temporal preparation relate to behavioral speeding effects. In fact, reflex amplitudes often correlate only weakly with RT (Requin et al., 1977; Semjen et al., 1973), and RT itself may be biased by eliciting reflexes during preparation. An exception was reported by Manning and Hammond (1990), who directly compared constant and variable FP conditions and found that constant FPs yielded smaller reflex amplitudes and shorter RTs than variable FPs, indicating stronger preparatory inhibition under higher temporal predictability. In a second experiment, they investigated preparation effects from target delivery to response onset. This time window can be segmented into a pre-motor phase, characterized by a relatively stable amplitude of elicited reflexes, and a motor phase, characterized by a sudden increase in reflex amplitude shortly before the overt response, presumably reflecting a response-related increase in spinal excitability. Crucially, the duration of this motor phase (but not the pre-motor phase) was influenced by FP duration and variability, and the pattern of results closely paralleled that obtained for RT.

Nevertheless, it must be noted that most reflex findings arise from simple RT tasks, which do not require response selection (Donders, 1969) and thus permit motor pre-programming, which may lead to an overestimation of the relevance of motor-level temporal preparation. In fact, under choice conditions or tasks requiring discrimination, limb-specific preparatory inhibition is often reduced or absent (Requin et al., 1977; Scheirs and Brunia, 1985; Semjen et al., 1973). Thus, while reflex studies support a motor contribution to temporal preparation, this may be limited to situations that allow advance motor programming and thus may not completely transfer to choice RT paradigms.

### Eye movements

A quite novel development in the study of temporal preparation concerns the impact of preparation on eye movements such as saccades and eye blink rate. A special focus has been placed on oculomotor inhibition of saccades during the foreperiod leading up to target presentation. Indeed, various studies have demonstrated this influence, typically showing stronger inhibition of saccades in a brief temporal window immediately preceding stimulus onset, when temporal preparation is high rather than low. For example, stronger oculomotor inhibition prior to visual target onset was observed when foreperiod duration was constant rather than variable within experimental blocks, with increasing constant foreperiod duration, and with decreasing variable foreperiod duration (Amit et al., 2019). Interestingly, stronger pre-target saccadic inhibition for constant vs. variable foreperiods was also observed for tactile (Badde et al., 2020) and auditory (Abeles et al., 2020) WS-target combinations, suggesting that preparation-related inhibition in the oculomotor system acts on a supramodal level. Trial-by-trial FP sequence also affects the oculomotor system, with weaker inhibition after long previous FPs (Tal-Perry and Yuval-Greenberg, 2023), and attentional re-orienting seems possible as well (Tal-Perry and Yuval-Greenberg, 2020). Complementing traditional foreperiod results, stronger saccadic suppression was observed for stimuli embedded within a rhythmic vs. arrhythmic temporal context (Dankner et al., 2017), and the interplay of hazard-based, uncertainty-based and instruction-based temporal preparation on saccade rate was investigated by Duyar and Carrasco (2025). In summary, preparation-related modulations of pre-stimulus saccadic inhibition resemble patterns typically observed in RT data, and interestingly, they can also be observed for stimuli that do not require any overt responses. This makes them a valuable tool to investigate on-line temporal preparation in the absence of overt responding (Tal-Perry and Yuval-Greenberg, 2021). Still, results seem somewhat mixed regarding the functional relevance of saccadic suppression. For example, (micro-)saccades occurring at or around the time of target presentation impaired discrimination performance for visual (Amit et al., 2019) as well as tactile targets (Badde et al., 2020), but not auditory ones (Abeles et al., 2020). Nonetheless, these findings unequivocally demonstrate that the oculomotor system is also affected by preparatory processes, with potential benefits for subsequent target processing.

### Transcranial magnetic stimulation

Transcranial magnetic stimulation (TMS) provides a noninvasive way to probe corticospinal excitability by inducing brief magnetic pulses over the motor cortex and recording motor-evoked potentials (MEPs) in corresponding muscles. Much like reflex studies, this method can be used to assess motor-level contributions to temporal preparation. For example, Hasbroucq et al. (1997) elicited MEPs in the Flexor Digitorum Superficialis, a forearm muscle that flexes the fingers, during a choice RT task with constant FPs (500 or 2,500 ms). When TMS coincided with the WS, MEP amplitudes did not differ between FPs. When delivered at target onset, however, MEPs were reduced after short FPs but unchanged after long ones. This decrease in excitability was interpreted as a marker of temporal preparation. A second experiment showed a progressive decline in MEP amplitude across the 500-ms FP, stabilizing approximately 167 ms before target onset, suggesting an inhibitory filtering mechanism that improves the signal-to-noise ratio for the forthcoming motor command. Later studies replicated and extended these findings, indicating that preparation reflects a dynamic interplay of inhibition and excitation within the motor cortex (Davranche et al., 2007; Sinclair and Hammond, 2008; Van Elswijk et al., 2007). For instance, corticospinal inhibition may co-occur with enhanced preparation-related cortical activation, and serve to suppress premature responses which might otherwise emerge (Davranche et al., 2007).

Direct comparisons of TMS-induced MEPs and H-reflexes during preparation (Hasbroucq et al., 1999a) revealed similar overall decreases but slightly different time courses, which is consistent with distinct sources of inhibition (presynaptic motor afferents for reflexes; cortical for MEPs). Because such inhibitory effects occur within the motor system and can be observed in choice RT tasks (Davranche et al., 2007; Hasbroucq et al., 1997, 1999a), they cannot be attributed solely to motor pre-programming (see also Hasbroucq et al., 1999b). A potential criticism is that TMS itself may disrupt preparation or act as an auxiliary WS, complicating the interpretation of RT data (Hasbroucq et al., 1999a). To avoid such online interference, Vallesi et al. (2007) applied offline TMS to reduce cortical excitability for several minutes. In a variable-FP paradigm, offline TMS over the right dorsolateral prefrontal cortex (rDLPFC) selectively reduced the variable-FP effect. In contrast, stimulation of the left dorsolateral prefrontal cortex or the right angular gyrus had no effect. Sequential FP effects, however, remained unchanged. TMS effects on saccadic latencies likewise suggest involvement of rDLPFC in the preparation of eye movements (Nagel et al., 2008). These findings implicate right prefrontal regions in monitoring temporal structure (Lewis and Miall, 2003; Rao et al., 2001), and indicate that variable FP and sequential effects may rely on distinct neural mechanisms, providing some evidence against simple conditioning accounts that posit a common origin (e.g., Los and Van Den Heuvel, 2001; Los et al., 2001).

In sum, TMS studies demonstrate that temporal preparation reduces corticospinal excitability, supporting a motor locus of preparation, consistent with findings from reflex studies. At the same time, evidence for prefrontal cortical involvement suggest that temporal preparation is not purely motoric and likely also engages higher-level pre-motor structures involved in temporal monitoring.

### Contingent Negative Variation

The results reviewed above concern effects of temporal preparation at rather peripheral motor levels (EMG, reflexes, MEPs, eye movements). A more direct view of cortical activity during preparation, with excellent temporal resolution, can be obtained via electroencephalographic (EEG) recordings. As raw EEG reflects a mixture of ongoing processes from different areas, rhythms, and artifacts, averaging time-locked segments to isolate specific event-related potentials (ERPs; for an overview, see Jäncke, 2005; Leuthold et al., 2004; Luck, 2005) has been especially useful to investigate processing during the FP and after target onset. An ERP component closely linked to temporal preparation was first described by Walter et al. (1964). They observed a slow negative potential developing between a WS and an imperative stimulus, but only when a response was required. This Contingent Negative Variation (CNV) diminished when WSs were not always followed by targets, and disappeared entirely when participants were informed that no further responses were required, yet it persisted if participants were instructed to respond at the expected imperative moment despite target absence. Thus, the CNV tracked the contingency between WSs and required responses, not merely the presence of a target. Although modulated by expectancy and voluntary movement preparation, Walter et al. also showed that CNV-like activity can accompany conditioned reflex responses, leading to its interpretation as a marker of motor preparation.

Later work revealed that the CNV is not unitary. With longer FPs, two components appear: an early CNV or orienting (O-) wave shortly after the WS, and a late CNV or expectancy (E-) wave just before the expected target (e.g., Gaillard, 1976; Loveless and Sanford, 1974). The early CNV reflects orienting to the WS and is sensitive to WS properties such as intensity, modality, and information content (e.g., Gaillard, 1976; Gaillard and Perdok, 1980; Loveless and Sanford, 1975). In contrast, the late CNV has been strongly linked to response preparation. This distinction was clearly demonstrated by Gaillard (1977), who orthogonally manipulated target probability (0%, 50%, and 90%) and response instructions (speed, accuracy, and delayed response). A late CNV developed when participants expected a target and were instructed to respond; yet, high target probability alone was insufficient when motor preparation was discouraged (delayed condition). In contrast, the early CNV appeared across all conditions, supporting the functional separation of the two sub-components.

Other studies confirmed the sensitivity of the late CNV to expectancy and response requirements (Besrest and Requin, 1973; McAdam et al., 1969; Trillenberg et al., 2000). In constant FP tasks, temporal preparation affects late CNV amplitude, which typically show decreasing amplitudes with increasing FP duration (Gaillard, 1976; Gaillard and Näätänen, 1973; Loveless and Sanford, 1975; McAdam et al., 1969; Müller-Gethmann et al., 2003). In variable FP paradigms, late CNV amplitude tracks the conditional probability of target onset, also mirroring typical RT effects. Specifically, the CNV amplitude increases with FP duration under aging distributions, remains stable under non-aging distributions, and peaks at the modal FP in Gaussian distributions (Trillenberg et al., 2000). Similarly, sequential effects—where current preparation depends on the previous trial's FP—appear in CNV amplitude (Los and Heslenfeld, 2005; Van der Lubbe et al., 2004).

In recent studies manipulating temporal cues, CNV amplitude typically is enlarged when the cue explicitly informs about the upcoming FP length, compared to neutral or invalid cues (Liu et al., 2025; Mento et al., 2015; Miniussi et al., 1999). Similarly, CNV amplitude is reduced if temporal expectations, based on blockwise manipulation of FP distribution, are violated (Jin et al., 2020). Additional evidence comes from paradigms inducing temporal regularities (i.e., regular rhythmic sequences of target stimuli), where CNV amplitude synchronizes with actual stimulus timing, and reflects expected temporal regularity even in case of occasional violations in temporal structure (Praamstra et al., 2006; Praamstra and Pope, 2007). Los and Heslenfeld (2005) further showed that explicit temporal cues modulate late CNV, yet do not eliminate sequential effects. This finding suggests that temporal preparation reflects an interplay of intentional cue-based strategies and unintentional, experience-dependent updating of temporal expectations.

Across these paradigms, the late CNV amplitude typically correlates reliably with RT (e.g., Gaillard and Näätänen, 1973; Los and Heslenfeld, 2005; McAdam et al., 1969; Trillenberg et al., 2000), reinforcing its interpretation as a marker of temporal preparation. Its distribution over centro–parietal scalp sites (e.g., Brunia and van Boxtel, 2001; Gaillard, 1976; Loveless and Sanford, 1974, 1975) and its resemblance to the readiness potential (LRP; Gaillard, 1980; Kornhuber and Deecke, 1965; Smulders and Miller, 2012, for a review; see also below) support a motor-system origin. Lateralization of the late CNV when response hands are known in advance further aligns it with pre-motor and motor cortical activity (Gaillard and Perdok, 1980).

However, this motor interpretation is not uncontested. The stimulus-preceding negativity (SPN)—a negativity preceding relevant stimuli without requiring motor responses—can overlap with late CNV (Brunia and van Boxtel, 2001). High-density EEG mapping studies implicate additional non-motor sources of the CNV, including anterior cingulate and dorsolateral prefrontal cortex (Dias et al., 2003), paralleling TMS evidence for prefrontal involvement in temporal preparation (e.g., Vallesi et al., 2007). Other work has tied CNV amplitude to non-motoric processes as effort (Wascher et al., 1996) and memory processes (Ruchkin et al., 1995).

In summary, the late CNV consistently varies with temporal preparation across a wide range of paradigms and correlates strongly with RT. It indexes that temporal predictions modulate preparatory states before target onset, and reflects preparation effects based on multiple sources of temporal structure, including time uncertainty, hazard functions, sequential statistics, and explicit cues, each of which may rely on partially distinct neural mechanisms (see Nobre and van Ede, 2018, 2023). While its properties—motor contingency, topographical distribution, and similarity to the readiness potential—largely support a motor locus of temporal preparation, recent evidence suggests that it may also index higher-order predictive processes beyond motor preparation, converging with the notion that temporal expectation may enhance perceptual and cognitive aspects of processing (e.g., Herbst and Obleser, 2019; Jin et al., 2020; Rohenkohl et al., 2012).

### Lateralized Readiness Potential

The notion that temporal preparation facilitates early processes is supported by studies employing the *Lateralized Readiness Potential* (LRP; see Kornhuber and Deecke, 1965). This potential is derived by measuring EEG activity over the left and right motor cortices and then subtracting the brain activity of the hemisphere ipsilateral to the response hand from that of the contralateral hemisphere. Thereby, activation specific to the response hand can be isolated, such that the emerging difference potential reflects the onset of hand-specific response preparation (e.g., De Jong et al., 1988; Gratton et al., 1988). Because the source of the LRP is presumably located in the primary motor cortex (Kristeva et al., 1991; Leuthold et al., 2004; Miller et al., 1992; Okada et al., 1982; Osman and Moore, 1993), the LRP has been used as a physiological marker of motor activation at a central level (for overviews, see Coles, 1989; Eimer, 1998; Smulders and Miller, 2012). Several studies have shown that when participants know in advance which hand will respond—via fixed response mappings, precues, or blocked instructions (De Jong et al., 1988; Gratton et al., 1988; Los and Heslenfeld, 2005; Van der Lubbe et al., 2004)—an LRP emerges already during the FP. This FP-LRP is sensitive to temporal preparation through explicit temporal cues, and both current and previous FPs modulate its amplitude (Los and Heslenfeld, 2005; Van der Lubbe et al., 2004).

In two-choice tasks in which no prior hand information is provided, the LRP develops only after target onset (e.g., Osman et al., 1995). Because hand selection cannot begin before stimulus identification, LRP onset marks the completion of preparatory processes preceding hand selection, enabling a bisection of RT into an early stimulus-locked (S-LRP) premotor interval and a late response-locked (LRP-R) motor interval (see also Mordkoff and Gianaros, 2000). Using Osman et al. (1995) method, Müller-Gethmann et al. (2003) varied the FP from 50 to 6,400 ms in a constant-FP paradigm and observed the typical RT pattern. RTs were shortest at medium FPs (200–400 ms) and became progressively slower as FPs increased. The S-LRP durations were strongly positively correlated with RT across FP conditions, indicating that most of the FP effect arises during early, stimulus-related processing. By contrast, LRP-R durations showed little or no relation to FP and did not correlate with RT. Overall, their findings suggest that WSs speed responses primarily by influencing early stimulus-related processes, rather than the later hand-specific motor preparation or execution stages, a result consistent with the early-onset model.

Extending this approach, Hackley et al. (2007) additionally analyzed the N2pc component (a lateralized posterior negativity that indicates visual target selection in bilateral displays, and thus, attentional selection after initial perceptual analysis is completed). Specifically, they used the latency of this component as an additional electrophysiological marker to divide RT into three temporal intervals (an early interval from stimulus onset to N2pc onset, a middle interval from N2pc onset to S-LRP onset, and a late interval from LRP-R onset to the response). They found that the FP effect was mainly restricted to the middle interval, suggesting that temporal preparation primarily speeds late perception, response selection, or early motor processes.

Comparable S-LRP effects have also been observed for expectations based on rhythmic vs. arrhythmic spatiotemporal motion (Rohenkohl and Nobre, 2011) and for trials with vs. without very short FPs (Hackley and Valle-Inclán, 1998, 1999), though these effects likely reflect arousal rather than temporal preparation (Hackley and Valle-Inclán, 2003).

The absence of preparatory modulation in the LRP-R interval seems to be at odds with previous studies (see above) suggesting pronounced effects within the motor system. However, as noted by Tandonnet et al. (2003, 2006), because an actual facilitation on the contralateral hemisphere—the one controlling the response—may be masked if the ipsilateral hemisphere shows no effect or even a reversed pattern. To address this problem, Kimura et al. (2025) employed surface Laplacian waves. Their analyses showed that FP-related modulations in motor preparation are more spatially focal than previously suggested, with short FPs producing a clearer, steeper buildup of contralateral motor-cortical activity. Their findings do not contradict the early-onset account; yet, they extend it by demonstrating that part of the FP effect also reflects changes in the contralateral motor readiness.

In sum, LRP-based RT bisection consistently shows robust temporal-preparation effects on the S-LRP interval (closely mirroring the results of Bausenhart et al., 2006, obtained using the PRP method for RT bisection), and thus imply that preparation speeds up premotor processing, while the LRP-R interval typically remains rather unaffected by temporal preparation. Yet, null effects in the LRP-R interval should be interpreted cautiously, and recent studies reopen the possibility that motor-related changes may also be reflected in the LRP component.

### Post-stimulus ERP components

Next to the LRP, a large number of EEG studies focused on *post-stimulus ERPs* to investigate the effects of preparation on the online processing of target stimuli. Various components of the ERP are conventionally named according to their polarity and relative latency (e.g., P1, N1, N2, P2, N400, P3; for overviews see Jäncke, 2005; Luck, 2005). Depending on their latency, scalp distribution and modulation by different manipulations, the functional significance of these ERP components ties them to specific processing stages: for example, P1, N1, and P2 are linked to sensory cortices and early perceptual encoding, N2 to attentional selection and detection of deviating or unexpected stimuli, and somewhat later perceptual processes, and P3 is often linked to more central, memory-related processes as context updating (cf. Jäncke, 2005).

Post-stimulus ERPs are thus especially useful to investigate whether temporal preparation modulates early stimulus processing. For example, Miniussi et al. (1999) used a task with speeded target detection and FP cues that were valid in 80% of trials. For short FPs, valid cues shortened P3 latency and increased its amplitude, while N2 was larger for invalid trials. P1 and N1 remained unaffected, suggesting a post-perceptual locus for temporal orienting. Similarly, Griffin et al. (2002) conducted two temporal orienting experiments with visual stimuli and valid cues (75% of trials). ERPs to nontargets after short FPs revealed that P1 and N1 were largely unaffected, N2 amplitude increased when cued to long FPs, and P3 latency shortened when cued to short FPs. It should be noted that, under conditions in which spatial orienting was presumably required, a diffuse N1 enhancement and a prolonged P3 latency were observed with short-FP cues, leaving somewhat unclear whether earlier processes might also show some preparation-based modulation.

Evidence for perceptual modulation emerges when temporal and spatial expectations interact.(Doherty et al. (2005) had participants track a moving ball with regular or irregular temporal and spatial trajectories. Temporal expectations alone attenuated posterior N1, while combined spatial and temporal expectations enhanced P1. Correa and Nobre (2008) replicated these effects using variable-paced occluded movements, showing N1 attenuation with longer FPs especially when the expected temporal regularity was violated (e.g., when the target appeared after an unexpected time). Again, N2 was attenuated, and P3 latency shortened with increasing temporal preparation. Rohenkohl and Nobre (2011) extended the tracking paradigm to include a more demanding discrimination task, and observed enhanced P1 and P3 amplitudes, along with modulating oscillatory alpha-band activity.

Subsequent research within the constant FP paradigm investigated the interplay of temporal and spatial factors more closely. Specifically, they assessed N2pc in visual search tasks involving multiple targets. In these studies, short compared to long FP duration typically shortened N2pc latency, indicating that preparation may increase the efficiency of visual selection of targets (Rolke et al., 2016; Seibold and Rolke, 2014) as well as for salient distractors (Balke et al., 2021), and this effect was more pronounced if target salience was low (Balke et al., 2022). Remarkably, also N1 was affected by foreperiod duration: its latency was reduced and its amplitude increased at shorter compared to longer FPs. Hence, these studies demonstrate clearly preparation-related enhancements of perceptual processes under conditions requiring visual search processes (see Hackley et al., 2007, for absence of a modulation of N2pc latency with a simpler display).

Some authors speculated that high perceptual demands may be crucial for observing early effects of temporal preparation. Correa et al. (2006a) had participants perform a difficult visual discrimination task in a temporal cueing paradigm. Consistent with the studies cited above, N2 was attenuated and P3 latency shortened after valid temporal cues; crucially, an enhanced P1 amplitude after valid cues was also observed. These results suggest that temporal orienting can enhance perceptual processing under demanding conditions even without spatial attention involvement.

Perceptual temporal preparation effects have also been observed in audition. Lange et al. (2003) presented auditory stimuli after short or long FPs and asked participants to selectively attend to one of the time points. They observed that temporal attention enhanced both N1 and P3 amplitude. Lange et al. (2006) and Sanders and Astheimer (2008) replicated this basic finding, and Lange and Röder (2006) extended it to tactile stimuli. Similarly, Herbst and Obleser (2019) reported that temporal predictability increases auditory N1 and P2 amplitude and decreases N1 latency, again indicating that temporal structure modulates early auditory perception. Overall, the rather consistent modulation of auditory ERPs seems to be in line with the idea that temporal information may be especially crucial for attentional selection in audition (Astheimer and Sanders, 2009; Tillmann and Lebrun-Guillaud, 2006), whereas visual processing relies primarily on spatial cues or spatio-temporal interactions unless tasks are highly demanding (Correa et al., 2006a; Griffin et al., 2002; Lange and Röder, 2006).

Despite the evidence for a preparation-based modulation of early ERPs, the interpretation of these findings can be complex. For example, N1 amplitude has shown enhancement (Lange et al., 2003; Sanders and Astheimer, 2008), no effect (Miniussi et al., 1999), or attenuation (Correa and Nobre, 2008; Doherty et al., 2005), and spatial factors of the stimulation seem to be relevant for consistent modulations of early ERPs. Moreover, early ERP modulations may reflect cortical excitability rather than target-specific processing speed (Lange and Röder, 2006), and (with the exception of difference waves as the LRP and the N2pc) may be influenced by overlapping late CNV waves (Hackley et al., 2007). In sum, however, temporal preparation consistently modulates N2 and P3 and can also affect earlier ERP components under certain perceptual or task demands, consistent with a pre-motor, perceptual locus of temporal preparation effects.

## Conclusion

Temporal preparation constitutes one of the most longstanding and robust research topics in experimental psychology. Early investigations relied almost exclusively on RT measures to study how advance temporal information shapes human performance. Across decades of research, FP effects have proven to be remarkably stable, replicable, and generalizable across tasks, modalities, and response requirements. This empirical robustness has made temporal preparation a cornerstone phenomenon for developing and testing models of human information processing and motor readiness.

Over time, the topic has increasingly attracted the attention of cognitive neuroscience. As a result, traditional RT measures have been complemented by neurophysiological indices such as electromyographic and electroencephalographic measures.^4^ These methods have provided converging evidence that temporal preparation is reflected in systematic changes in central and peripheral processes prior to and following target onset, thereby strengthening and refining inferences drawn from behavioral data alone. There is now convincing evidence that various cognitive processes can benefit from temporal processing, including perceptual and motor systems, and sometimes even more central decisional processes. The specific loci of temporal preparation effects appear to be strongly task-dependent (e.g., perceptual for perceptually demanding tasks, motoric for complex movements), but also influenced by the characteristics of the preparatory processes themselves. For instance, the time available for preparation, specific demands of the task (e.g., simple vs. complex decisions), and complex interactions with other factors affecting predictability (e.g., spatial or event uncertainty) may significantly shape how temporal preparation affects behavior and neural activity.

Notably, within various of the motor-related measures (reflex amplitude, TMS, saccade rate) reviewed above, inhibitory modulations based on temporal preparation have been consistently identified. As recently suggested by Greenhouse et al. (2015) in the context of action selection, such global preparatory inhibition may not only serve to prevent premature responding, but also gain functional relevance by reducing noise and modulating gain related to selection and initiation of responses. Even though it is unclear whether similar mechanisms underlie the case of temporal preparation, in any case, inhibition seems to be a key signature of preparatory adjustments and therefore should be considered a cornerstone for theoretical developments.

The widespread consequences of temporal prepararation throughout the cognitive system, from signs of global inhibitory preparatory activity during the preparation phase to facilitation of target processing in pre-motor and motor stages after stimulus onset, seem difficult to reconcile with a single theory of preparation. To date, the most comprehensive framework is the formal model of temporal preparation (fMTP), which emphasizes associative learning between temporal intervals and preparatory states defined by an interplay of inhibition and activation. While this associative account is highly successful in capturing a broad range of FP phenomena, an open question remains whether this is sufficient on its own or whether higher-order cognitive processes–such as executive control, strategic expectancy, or goal-dependent modulation–also contribute to shaping preparatory states, which would imply the necessity of multicomponent accounts of temporal preparation (for example, see Vallesi et al., 2007). In order to gain such a more complete understanding, it seems beneficial to systematically study and compare different sources of temporal preparation. A notable recent development enabling such systematic study is the TEP-Task (Capizzi et al., 2026), which allows for simultaneous assessment of the most common temporal preparation (FP, sequential, orienting, and rhythmic) effects within a single experimental session. Moreover, accounts that predict preparatory effects in specific processing systems, such as the Motor Readiness (Mattes and Ulrich, 1997; Näätänen, 1971) and the Early Onset Model (Rolke and Hofmann, 2007) have received ample support from different lines of research, suggesting that preparation effects on target processing may emerge through multiple underlying mechanisms, influencing information accumulation as well as motor activity. In our opinion, a future challenge will be to develop straightforward models to determine which mechanisms may become relevant under which conditions, rather than pitting seemingly competing, yet not mutually exclusive, mechanisms against each other.

Understanding FP effects is important not only for theoretical reasons, but also because temporal preparation is ubiquitous in experimental paradigms and everyday behavior. In basically any laboratory task with repeated trials, pre-stimuli (i.e., a fixation cross) are presented, or a temporal relationship between subsequent trials exists (either variable or constant intertrial intervals). Necessarily these factors will affect the state of temporal preparedness, even when temporal preparation is not the primary object of study, and this may add to or interact with experimenter's manipulations of interest. Recent work has demonstrated, for example, that variable FP duration modulates stimulus-response bindings (Schmalbrock and Frings, 2022), and cue-stimulus or response-stimulus interval duration is well known to be an important modulator of cognitive control processes in task switching and conflict tasks (e.g., Duthoo et al., 2014; Meiran, 1996)—highlighting that temporal factors may influence core mechanisms of action control and learning.

Of course, also beyond the laboratory, anticipation and readiness for perception and action play a fundamental role in many aspects of everyday life, from the preparation of movements in sports (Dalmaijer et al., 2015; Ramsay et al., 2025), driving behavior (Näätänen and Summala, 1976; Stahl et al., 2014), to the timing of turn-taking in communication (Magyari et al., 2017)—but, especially given the longstanding research tradition on temporal preparation, surprisingly little research has been devoted to examining the role of temporal predictions in such fields more closely.

Clearly, despite the longstanding tradition of studying temporal preparation effects, ongoing research efforts are needed to shed light on these issues (see also Box 2). In sum, advancing our understanding of temporal preparation not only informs basic theories of perception, cognition, and action, but also provides insight into a pervasive mechanism that enables temporally adaptive behavior in dynamic environments.

Box 2 **Research questions.**

1. Can preparatory mechanisms underlying constant FPs, variable FPs, and temporal cues be dissociated or are temporal preparation effects in various paradigms based on a universal mechanism and functionally equivalent?
2. Inhibitory adjustments seem to play a major role, especially in motor-related temporal preparation effects. But can such modulations also successfully predict modulations of earlier preparation effects, for example, sensory gain?
3. What is the relation of temporal preparation and alerting? Can they be dissociated and if so, to what extent have existing studies confounded their effects? If not, can a single mechanism or unified framework account for non-linearity of effects?
4. Preparation seems to flexibly enhance processing at various stages of the cognitive system; potentially depending on task characteristics and processing demands. Yet, process-oriented models about mechanisms that would allow us to make testable predictions about this relationship are currently missing.
5. Are higher-order cognitive functions involved in temporal preparation beyond simpler associative processes—and if so, under which conditions? Further extension of recent research efforts on the role of motivation and goal-directed action planning, control processes and criterion setting, conceptual knowledge, cognitive capacity and working memory processes in temporal preparation could help clarify these issues.

## Footnotes

1 Here and in the following, we use the term “temporal preparation” as a theory-neutral description of performance changes that arise when the timing of an event is more or less certain. It should not be taken to imply any specific claim about whether preparatory processes are intentional or automatic, nor about the underlying mechanisms that produce them. See below for different theoretical stances on the origins of temporal preparation.

2 It should be noted that researchers also have successfully studied the preparatory processes underlying temporal and event uncertainty in combination, for example, by deliberately confounding target type and foreperiod duration, or studying the interplay of foreperiod and event repetitions vs. switches (e.g., Langner et al., 2018; Schröter et al., 2015; Thomaschke and Dreisbach, 2013, 2015; Wagener and Hoffmann, 2010).

3 A recurring distinction in this context is that between “pre-motor” and “motor” processes. However, what counts as pre-motor vs. motor depends on the method used to define the boundary. For instance, in EMG studies, “pre-motor” typically refers to the period preceding muscular activation. In RT studies, it may be used to denote processes occurring before movement onset, or in earlier stages up to response selection, prior to motor programming. In studies examining reflex amplitudes, the distinction may instead be based on abrupt changes in reflex responsiveness, reflecting the release of corticospinal inhibition during the foreperiod (FP). We tried to clarify how these specific distinctions are made within the following sections, but we advise caution that conclusions based on these definitions may not be directly comparable across different methodologies and measures.

4 Even though largely beyond the scope of the present review, we would like to acknowledge that advanced neuroscientific methods, including functional neuroimaging techniques, analyses of cortical oscillations, and single-cell recordings, contributed immensely to our understanding of temporal preparation and have further substantiated temporal preparation as a fundamental, complex phenomenon underlying cognitive processing (e.g., Cravo et al., 2013; Ghose and Maunsell, 2002; for excellent overviews, see Coull, 2004; Nobre and van Ede, 2018).
